# Factors Associated with Patient and Provider Delays for Tuberculosis Diagnosis and Treatment in Asia: A Systematic Review and Meta-Analysis

**DOI:** 10.1371/journal.pone.0120088

**Published:** 2015-03-25

**Authors:** Jing Cai, Xianhua Wang, Aiguo Ma, Qiuzhen Wang, Xiuxia Han, Yong Li

**Affiliations:** 1 The College of Public Health, Qingdao University, Qingdao, Shandong Province, PR China; 2 The College of Public Health, Beijing University, Beijing, PR China; Beijing Institute of Microbiology and Epidemiology, CHINA

## Abstract

**Background:**

Delays in tuberculosis (TB) diagnosis and treatment is a major barrier to effective management of the disease. Determining the factors associated with patient and provider delay of TB diagnosis and treatment in Asia may contribute to TB prevention and control.

**Methods:**

We searched the PubMed, EMBASE and Web of Science for studies that assessed factors associated with delays in care-seeking, diagnosis, or at the beginning of treatment, which were published from January 1992 to September 2014. Two reviewers independently identified studies that were related to our meta-analysis and extracted data from each study. Independent variables were categorized in separate tables for patient and provider delays.

**Results:**

Among 45 eligible studies, 40 studies assessed patient delay whereas 30 assessed provider delay. Cross-sectional surveys were used in all but two articles, which included 17 countries and regions. Socio-demographic characteristics, TB-related symptoms and medical examination, and conditions of seeking medical care in TB patients were frequently reported. Male patients and long travel time/distance to the first healthcare provider led to both shorter patient delays [odds ratio (OR) (95% confidence intervals, CI) = 0.85 (0.78, 0.92); 1.39 (1.08, 1.78)] and shorter provider delays [OR (95%CI) = 0.96 (0.93, 1.00); 1.68 (1.12, 2.51)]. Unemployment, low income, hemoptysis, and positive sputum smears were consistently associated with patient delay [ORs (95%CI) = 1.18 (1.07, 1.30), 1.23 (1.02, 1.49), 0.64 (0.40, 1.00), 1.77 (1.07, 2.94), respectively]. Additionally, consultation at a public hospital was associated with provider delay [OR (95%CI) = 0.43 (0.20, 0.91)].

**Conclusions:**

We propose that the major opportunities to reduce delays involve enabling socio-demographic factors and medical conditions. Male, unemployed, rural residence, low income, hemoptysis, positive sputum smear, and long travel time/distance significantly correlated with patient delay. Male, long travel time/distance and consultation at a public hospital were related to provider delay.

## Introduction

The severe epidemic situation of tuberculosis (TB) in Asia is well documented, and TB prevention and control is a top priority for public health. TB can cause enormous social and economic disruption, cause a series of serious consequences [1], and hamper a nation’s development [2]. In 2012, there were an estimated 8.6 million new TB cases and 1.3 million TB deaths [3]. Of the 22 high-TB burden countries, which account for approximately 80% of the world’s TB cases [4], almost all are developing countries where one-quarter of the world’s poorest populations lives. Southeast Asia and the Western Pacific region collectively accounted for 58% of the world’s TB cases in 2012, and India and China had the largest number of cases (26% and 12% of the global total, respectively). Moreover, as reported by the World Health Organization (WHO) [3], approximately 75% of the estimated 2.9 million cases were either not diagnosed or diagnosed but not reported to national tuberculosis programs in 12 countries, including India (31% of the global total), South Africa, Bangladesh, Pakistan, Indonesia, China, Democratic Republic of the Congo, Mozambique, Nigeria, Ethiopia, the Philippines, and Myanmar.

TB is preventable if people can access health care for prompt diagnosis and treatment. However, diagnosis and treatment delays are inevitable, and can be categorized as patient delays, provider delays (including health system or doctor delays), diagnostic delays (sometimes the sum of patient and provider delays), and treatment delays. Delays are significant regarding disease prognosis and complications at the individual level, as well as for transmission within the community, the reproductive rate of the TB epidemic [5], and the possibility of higher mortality [6, 7]. Delays can affect the rate of infection among close contacts [8], and the emergence of multidrug-resistant TB [9]. Thus, identifying when delays occur and the factors that correlate with different types of delay can aid tuberculosis control programs and help medical providers improve diagnosis and treatment efforts [10]. TB control programs must identify the reasons for delays in order to improve the control strategies.

It has been reported that one-third of the world’s TB burden, or approximately 4.9 million prevalent cases, are found in Southeast Asia [11]. Although similar systematic reviews have addressed delays in diagnosis and treatment and have reported the average (median or mean) total diagnosis delay, patient delay, and health system delay as 25–185 days, 4.9–162 days, and 2–87 days, respectively, for both low and high income countries [12], as well as India [13], no study has focused solely on Asia. One meta-analysis [14] described factors associated with patient and diagnostic delays in Chinese TB patients, and another analyzed factors associated with patient and health care system delays for TB in sub-Saharan African countries [15]. Thus, a systematic analysis of factors associated with patient and provider delays for TB that focuses on Asian countries is necessary to help design interventional studies suited to the culture, economic, and co-morbid patterns of this region. We sought studies conducted in adults in Asian countries to identify factors correlated to patient and provider delays in TB diagnosis. Our results may contribute to the practical work of TB prevention and control.

## Methods

### Inclusion and exclusion criteria

We used the following inclusion criteria: (1) studies that included TB patients (or suspected TB patients) in Asia; (2) studies that investigated factors that correlated with patient or provider delays in TB diagnosis and treatment; (3) studies designed as observational studies (cross-sectional, case—control, or cohort studies); and (4) studies that provided enough information to calculate the odds ratio (OR) and 95% confidence intervals (CI). If there was more than one article with the same population or data, the study with the largest population and most complete data was selected.

Our exclusion criteria were: (1) studies that included other diseases; (2) studies that did not investigate the associated factors; (3) duplicate studies; and (4) review articles, case reports, or comments without original data.

Two authors (JC, XW) independently screened the titles and abstracts of the studies that were initially included to assess their eligibility based on the inclusion and exclusion criteria. When there was a disagreement regarding the qualifications of the studies, the reviewers discussed whether the studies should be included until a consensus was reached.

### Search strategy

Studies published in English from January 1992 to September 2014 that described original research were searched worldwide [16]. Eligible studies involved at least one factor related to delays in care-seeking, diagnosis, or at the beginning of treatment. We searched the PubMed, EMBASE, and Web of Science databases from January 1992 to September 2014. The search was limited to studies in humans. Search terms, including ‘tuberculosis’, ‘patient delay’, ‘provider delay’, ‘diagnosis delay’, ‘treatment delay’, ‘health system delay’, ‘Asia’ and countries in Asia, were searched in the title and abstract. We reviewed the references of the included studies and reviews. If it was unclear whether studies were eligible, the full texts were reviewed. The PubMed search strategy is shown in Table 1.

**Table 1:** PubMed databases search terms and strategy.

	Search terms
1	provider delay
2	diagnosis late
3	delay treatment
4	patient delay
5	health seeking OR care-seeking
6	treatment seek
7	case detection OR case finding OR case diagnosis
8	delay present OR delay diagnosis OR diagnostic errors OR early diagnosis OR undiagnosed
9	1 OR 2 OR 3 OR 4 OR 5 OR 6 OR 7 OR 8
10	Tuberculosis
11	Asia OR China OR Hong Kong OR Macao OR Taiwan OR Japan OR Korea OR Mongolia OR myanmar OR Cambodia OR Indonesia OR Malaysia OR the Philippines OR Singapore OR Thailand OR Vietnam OR Bangladesh OR India OR maldives OR Nepal OR Pakistan OR Afghanistan OR Iran OR Israel OR Saudi Arabia OR Turkey
12	9 AND 10 AND 11

### Quality assessment

We rated the quality of all included studies using the Newcastle Ottawa Quality Assessment Scale (NOS) for non-randomized studies in meta-analyses [17] (Table 2).

**Table 2:** Characteristics of the 45 included studies.

Author, year	Location	Study design	Sample	NOS score
Wang W (2007)[18]	China	Cross sectional study	n = 222; newly PTB patients; 76% aged 20–40 years; sex not recorded	4
Leung EC (2007)[19]	Hongkong	Retrospective study	n = 1249; pulmonary TB; 68.4% male; 47.7% were aged ≥60 years	4
Huong NT (2007) [20]	Vietnam	Cross sectional study	n = 2093; Smear-positive PTB patients; 71% male; 22.6% were ≥65 years	5
Enkhbat S (1996)[21]	Mongolia	Cross sectional study	n = 107; ≥16 years TB patients; 54% male; Mean age = 33 years	4
Yamasaki-Nakagawa M (2001) [22]	Nepal	Cross sectional study	n = 390; new TB cases + began to receive Direct Observation Therapy, Short-course; 67.9% male; Median age in men = 37 years, in women = 30 years	5
Chiang CY (2005)[23]	Taiwan	Cross sectional study	n = 206; new sputum-positive PTB patients; 69.4% male, Mean(range) age = 59.2 (17–91) years	5
Rojpibulstit M (2006) [24]	Thailand	Cross sectional study	n = 192; newly diagnosed smear-positive and smear-negative PTB patients; 75% male; Mean (SD, range) = 43.3 (16.2, 15–83) years	5
Li X (2012)[25]	China	Cross sectional study	n = 323; >18 years + migrant + new PTB; 63.8% male; median (inter-quartile range) = 30 (24–38) years	5
Lock WA (2011)[26]	Indonesia	Cross sectional study	n = 194; TB suspects; 54% male; Mean (SD) = 39 (14.1) years	5
Zhou C (2012)[27]	China	Cross sectional study	n = 314; TB patients being treated or had finished standard treatment within 6 months; 63.4% male; age mean (range, median) = 31.8 years (15–70, 28)	5
Chang CT (2007)[28]	Malaysia	Cross sectional study	n = 316; new smear-positive PTB patients + adult; 61.1% male; age mean (range, median) = 44.2 (15–7, 45) years	5
Lin X (2008)[29]	China	Cross sectional study	n = 10356; smear-positive PTB cases; 65.5% male; Median (IQR) = 38 (27–54) years	5
Basnet R (2009) [30]	Nepal	Cross sectional study	n = 307; new TB patients; 59.0% male; 70.7% 15–54 years	5
Guneylioglu D (2004) [31]	Turkey	Cross sectional study	n = 204; smear-positive PTB + hospitalized patients; 59.8% male; Mean age = 33 years	4
Hoa NB (2011) [32]	Vietnam	Cross sectional study	n = 7040; TB suspects + >15 years; 62.9% male; Mean age (SD) = 48.9 (0.3) years	5
Xu B (2007) [33]	China	Cross sectional study	n = 1204; TB suspects; 61.6% male; 40–59 years 36.0%	4
Ahmad RA (2013) [34]	Indonesia	Cross sectional study	n = 746; TB suspects + ≥15 years; 38% male; ≥60 years 45.4%	4
Phoa LL (2005) [35]	Singapore	Cross sectional study	n = 375; adult PTB patients (sputum culture-positive and cough); M/F ratio 3:1, Median (range) = 49 years (15–90 years)	6
Xu X (2013) [36]	China	Cross sectional study	n = 4677; suspected TB cases; 64% male; 15–30 years 60.3%	5
Rundi C (2011) [37]	Malaysia	Cross sectional study	n = 296; smear-positive TB patients + ≥18 years; age and sex were not presented	6
Zhao X (2014) [38]	China	Cross sectional study	n = 819; TB patients + >15 years; 72.5% male; Median (range) = 53 (15–90) years	5
Cheng G (2005) [39]	China	Cross sectional study	n = 190; smear positive PTB patients; 65.3% male; Mean age (SD) = 48.1 (16.6) years	5
Tobe RG (2013) [40]	China	Cross sectional study	n = 314; smear positive PTB patients; 63.4% male; age was not presented	6
Choudhari M (2012) [41]	Nepal	Cross sectional study	n = 215; PTB patients; 65.6% male; Mean age = 39.6 years	6
Shu W (2014) [42]	China	Cohort study	n = 260; active TB patients; age and sex were not presented	7
Ngamvithayapong J (2001) [43]	Thailand	Cross sectional study	n = 557; smear positive PTB patients + >15 years; sex was not presented; Median (range) = 37 (29–52) years old	5
Selvam JM (2003) [44]	India	Cross sectional study	n = 667; new adult TB patients; 73% male; Mean age = 43 years	5
Chen HG (2014) [45]	China	Cross sectional study	n = 1126; PTB patients; age and sex were not presented	6
Kelkar-Khambete A (2008) [46]	India	Cross sectional study	n = 117; new sputum-positive TB patients; 60% male; 15–44 years 82%	5
Ayé R (2010) [47]	Tajikistan	Cross sectional study	n = 168; new PTB patients + ≥15 years; age and sex were not presented	4
Tobgay KJ (2006) [48]	India	Cross sectional study	n = 323; TB patients cough for ≥3 weeks; 58.8% male; Median = 30 years	6
Rumman KA (2008) [49]	Jordan	Cross sectional study	n = 1544; TB suspects + >15 years; 57.3% male; >35 years 62.6%	6
Wang Y (2008) [50]	China	Cross sectional study	n = 1005; adult suspected TB cases + >15 years; 54.1% male; ≥60 years 41.4%	6
Mor Z, (2013) [51]	Israel	Cross sectional study	n = 1005; adult suspected TB cases + >15 years; Citizen: 60.8% male; Mean age (SD) = 53.7 (19.7) years; Migrant: 45.5% male; Mean age (SD) = 35.0 (9.8) years	5
Rifat M (2011) [52]	Bangladesh	Cross sectional study	n = 7280; new smear-positive PTB patients; age and sex were not presented	5
Bai LQ (2004) [53]	China	Cross sectional study	n = 318; smear-positive PTB patients + ≥15 years; 72.0% male; Mean age (SD) = 38.2 (12.0) years	6
Tamhane A (2012) [54]	India	Cross sectional study	n = 150; newly smear-positive PTB patients + ≥18 years; 50% male; Median = 30 years	5
Sabawoon W (2012) [55]	Afghanistan	Cross sectional study	n = 122; smear-positive PTB patients + ≥15 years; 45.9% male; Mean age (SD) = 42.2 (17.7) years	4
Karim F (2007) [56]	Bangladesh	Cross sectional study	n = 1000; smear-positive PTB patients; 50% male; Mean age = 37.7 years	5
Thakur R (2010) [57]	India	Cross sectional study	n = 234; smear-positive PTB patients; 66.7% male; ≥35 years 50.9%	5
Lönnroth K (1999) [58]	Vietnam	Cross sectional study	n = 422; new TB patients + ≥15 years; 70.4% male; 51.1% were aged 15–34	4
Rajeswari R (2002) [59]	India	Cross sectional study	n = 531; new smear- positive TB patients; 72% male; 62%<45 years	5
Lin HP (2009) [60]	Taiwan	Cross sectional study	n = 78118; new PTB patients; 69.4%were male; 42648 (54.6%) patients were aged over 65 years	5
Wang WB (2006) [61]	China	Cross sectional study	n = 493; newly TB patients; 72.4% male; 40–60 years 38.1%	4
Lin CY (2010) [62]	Taiwan	Cross sectional study	n = 193; culture-proven, new hospitalized PTB patients; 67.9% male; Mean age (SD) = 63.47 (18.04) years	5

### Data extraction

Two authors (JC, XW) independently extracted the data from the eligible studies. Differences were resolved by discussion between the reviewers to reach a consensus. Available information was extracted from each study including the authors, publication year, location, starting points and endpoints of delays, study design, method of data collection, characteristics of samples, measurements of the dependent variables, data source, and conclusions.

### Statistical analyses

We summarized related characteristics and independent variables from the 18 selected studies using separate tables for patient and provider delays. The overall effect of factors related to delays was estimated in more than two studies by conducting a meta-analysis based on ORs and 95% CIs. If ORs and 95% CIs were not reported in the original article but sufficient data were provided, we used the calculator in Review Manager Software 5.2 (Cochrane Collaborative, Oxford, United Kingdom) to calculate ORs and 95% CIs. We used Q tests and the I-squared statistic [I^2^ = 100% × (Q-df)/Q] to quantify heterogeneity. Because of the small number of studies and the small sample size of some of the included studies, we used a P-value of 0.10 and an I^2^ of 50% to determine statistical significance. If the P-value of the Q statistic ≥0.10, or if P <0.10 but I^2^ ≤50%, we used fixed effect models; otherwise, random effect models were used.

## Results

### Identification of eligible studies

**Fig 1:**
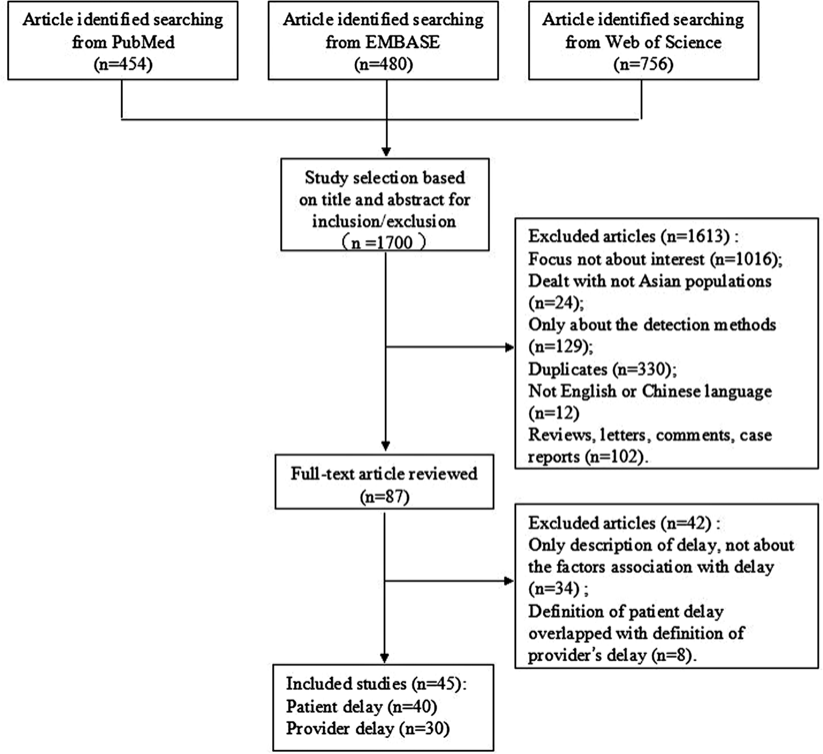
Flow Chart of literature search for studies on the patient delay and provider delays. This figure is a description of the full search process.

There were 1700 articles regarding factors associated with patient (care-seeking) delays or provider (health-system, doctor, or diagnostic) delays identified from the databases. We excluded 1613 articles after browsing the titles and abstracts, primarily because they did not have a dependent relationship with the topic. In addition, we also excluded reviews, letters, comments, or case reports. After searching 87 full texts, another 34 studies were excluded because although their initial aim focused on delays, correlated factors were not discussed. Eight articles were excluded because the endpoint of patient delay was the start of treatment, which overlaps with the definition of provider delay. Finally, 45 studies were included in this meta-analysis (Table 2). Fig. 1 shows a flow chart of the literature search for studies regarding patient and provider delays (Fig. 1).

### Study characteristics

The included articles were published between 1997 and 2014 (Table 3). The 45 articles covered 17 countries and regions, including mainland China (k = 14), India (k = 6), Vietnam (k = 3), Nepal (k = 3), Taiwan (k = 3), Indonesia (k = 2), Malaysia (k = 2), Thailand (k = 2), Bangladesh (k = 2), Turkey (k = 1), Mongolia (k = 1), Hong Kong (k = 1), Singapore (k = 1), Tajikistan (k = 1), Jordan (k = 1), Israel (k = 1), and Afghanistan (k = 1). Sample sizes in the studies ranged from 107 to 78,118. All studies except six conducted patient interview surveys (cross-sectional or cohort design), and used a consecutive method of patient sampling. In all the studied samples, the proportion of male patients was greater than 50%. Most studies were conducted in both rural and urban areas (k = 37). Finally, there were 41,185 TB cases on patient delay and 103,427 TB cases on provider delay.

**Table 3:** Factors assessed for association with patient delay (k = 40).

Citation	Delay = 1st sx to (interval)	Male sex	Old age	Education	Unemployed	Rural residence	Income low level	Hemoptysis	Cough	Positive sputum smear	Chest radiographs	Consult with a public hospital	Long travel time/distance
Wang W (2007)[18]	1st consult (21 days)	x	x	x	x	—	↑	↓	—	—	x	—	—
Leung EC (2007)[19]	1st consult (20days)	—	—	—	↑	—	—	↓	—	—	—	—	—
Huong NT (2007) [20]	1st consult (6 weeks)	x	x	—	—	↑	—	—	—	—	—	x	—
Enkhbat S (1996)[21]	1st consult (1 month)	x	x	—	—	x	x	—	—	—	—	x	—
Yamasaki-Nakagawa M (2001)[22]	1st consult (1 months or 7 months)	—	—	—	—	—	—	—	—	—	—	—	—
Chiang CY (2005)[23]	1st consult (7 days)	x	↑	—	—	—	—	x	x	x	x	x	—
Rojpibulstit M (2006)[24]	1st consult (interval not recorded)	x	x	—	—	—	—	—	—	x	—	—	—
Li X (2012)[25]	1st consult (10 days)	x	x	—	x	—	—	↓	—	—	—	—	—
Lock WA (2011) [26]	1st consult (2weeks)	x	x	x	—	x	x	—	—	—	—	↑	↑
Zhou C (2012)[27]	1st consult (14 days)	x	x	x	—	x	↑	—	—	—	—	—	↑
Chang CT (2007)[28]	1st consult (30days)	↓	—	—	—	—	x	—	—	—	—	—	—
Lin X (2008)[29]	1st consult (60 days)	—	↓	—	x	—	↓	—	—	—	—	—	—
Basnet R (2009) [30]	1st consult (30 days)	x	x	x	—	—	x	—	—	↑	—	—	x
Guneylioglu D (2004) [31]	1st consult (30 days)	x	x	x	—	x	↑	↓	↓	—	—	—	—
Hoa NB (2011) [32]	1st consult (interval not recorded)	↓	—	—	—	↓	x	—	—	—	—	—	—
Xu B (2007) [33]	1st consult (interval not recorded)	x	x	x	—	—	x	—	—	—	—	—	—
Ahmad RA (2013) [34]	1st consult (interval not recorded)	↓	—	—	x	—	—	—	—	—	—	—	—
Phoa LL (2005) [35]	1st consult (4 weeks)	x	↓	—	x	—	—	—	—	↑	—	—	—
Xu X (2013) [36]	1st consult (3 weeks)	↓	x	—	—	—	—	—	—	↑	—	—	—
Rundi C (2011) [37]	1st consult (30 days)	x	x	—	—	x	x	—	—	—	—	x	x
Zhao X (2014) [38]	1st consult (30 days)	↓	x	x	x	—	x	—	—	—	—	—	x
Cheng G (2005) [39]	1st consult (interval not recorded)	x	x	↓	—	—	x	—	—	—	—	x	x
Tobe RG (2013) [40]	1st consult (2 weeks)	x	—	x	—	x	↑	—	—	—	—	—	↑
Choudhari M (2012) [41]	1st consult (30 days)	x	↑	—	x	x	x	—	—	—	—	—	↑
Shu W (2014) [42]	1st consult (interval not recorded)	—	x	—	—	—	—	—	—	—	—	—	—
Ngamvithayapong J (2001) [43]	1st consult (21 days)	x	x	x	—	—	—	—	—	—	—	—	—
Selvam JM (2003) [44]	1st consult (28 days)	x	x	x	x	—	—	—	—	—	—	x	x
Chen HG (2014) [45]	1st consult (30 days)	x	↑	—	—	—	—	—	—	↑	Cavity:↑	—	—
Kelkar-Khambete A (2008) [46]	1st consult (21 days)	x	x	x	—	x	x	—	—	—	—	—	—
Ayé R (2010) [47]	1st consult (interval not recorded)	x	x	—	—	x	—	—	—	—	—	—	—
Tobgay KJ (2006) [48]	1st consult (30 days)	x	x	—	—	—	—	—	—	—	—	↑	—
Rumman KA (2008) [49]	1st consult (3 weeks)	↓	↑	↑	x	↑	↑	↑	—	—	—	—	x
Wang Y (2008) [50]	1st consult (2 weeks)	↓	↓	↓	x	—	↓	—	—	—	—	—	—
Mor Z (2013) [51]	1st consult (25 days)	x	x	—	x	—	—	—	—	↑	—	—	—
Rifat M (2011) [52]	1st consult (4 weeks)	x	x	—	—	↑	—	—	—	—	—	—	—
Bai LQ (2004) [53]	1st consult (14 days)	—	—	—	—	—	—	↓	—	—	—	—	—
Tamhane A (2012) [54]	1st consult (20 days)	—	—	—	x	—	—	x	—	—	—	—	—
Sabawoon W (2012) [55]	1st consult (interval not recorded)	—	—	—	—	—	—	—	—	—	—	↓	↑
Karim F (2007) [56]	1st consult (interval not recorded)	x	x	—	—	—	—	—	—	—	—	—	—
Thakur R (2010) [57]	1st consult (33.5 days)	x	x	x	—	x	↑	—	—	—	—	↑	—

x. Assessed but P ≥ 0.05

↓. Negatively correlated with delay, P < 0.05

↑. Positively correlated with delay, P < 0.05

—. Not assessed.

### Patient delay studies

Patient delay was measured using patients’ recall of first TB symptoms as the starting point, (usually described as the onset of a persistent cough) and the date of the first health facility consultation as the endpoint. All studies measured delay as a dichotomous variable, typically based on a median split from 7 to 60 days. Nearly all studies reported their definition of delay; however, some (k = 9) did not mention the specific interval of delay.

#### Factors assessed

Forty studies assessed associated factors, including patient socio-demographic characteristics, TB-related symptoms, medical examinations, and conditions of seeking medical care (Table 3). Socio-demographic characteristics included gender, age, education, occupation, rural residence, and income level. Thirty-three studies analyzed the relationship between male sex and delay, and seven showed male sex negatively correlated with delay. Thirty-one studies evaluated the role of age in delay, and 13 studies addressed unemployment and patient delay. Education level was discussed in 14 studies. Fourteen studies reported correlations between rural residence and patient delay. Nineteen studies paid attention to income level. TB symptoms were associated with patient delay in eight studies, in which cough was analyzed as the first symptom (k = 2) and hemoptysis (k = 8) was associated with patient severity. Positive sputum smears and chest radiographs were taken at the first consultation in seven and three studies, respectively. Consulting a public hospital was associated with delay in ten studies. Long travel time or distance to the first healthcare provider was investigated in 11 studies.

#### Meta-analysis

**Fig 2:**
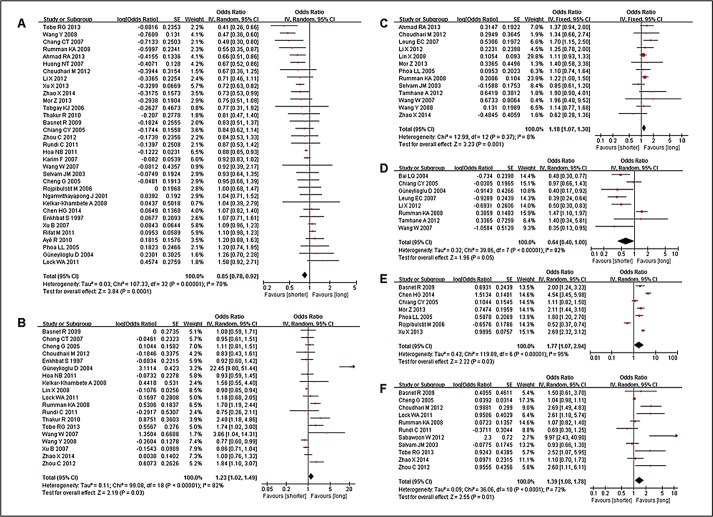
Forest plots of meta-analysis of factors associated with patient delay. This figure shows forest plots for the meta-analysis of five factors associated with patient delay. OR and 95% CI for each factor are given. Notes: A- Male sex; B- Low income; C- Unemployed occupation; D- Haemoptysis; E- Smear positive; F- Long travel time/distance to the first healthcare provider.

Male, unemployed, low income level, hemoptysis, positive sputum smears, chest radiographs, and long travel time or distance to the first healthcare provider all significantly correlated with patient delay (Table 4). Male and hemoptysis were negatively associated with patient delay, whereas the other three factors were positively associated (P<0.05). There were no significant relationships between delay and older age, education level, rural residence, chest radiographs, or consultation with a public hospital (Fig. 2). Cough and other factors could not be evaluated due to a lack of data in the studies.

**Table 4:** Results of meta-analyses and test for heterogeneity of patient delay.

Variable	k	OR (95% CI) ^a^	Heterogeneity between studies			Test for overall effect (P)
			Q statistic	P-value	I^2^	
Male sex	33	0.85 (0.78, 0.92)	107.33	<0.00001	70%	0.0001
Unemployed	13	1.18 (1.07, 1.30)	12.99	0.37	8%	0.001
Income (Low)	19	1.23 (1.02, 1.49)	99.08	<0.00001	82%	0.03
Haemoptysis	8	0.64 (0.40, 1.00)	39.86	<0.00001	82%	0.05
Positive smear	7	1.77 (1.07, 2.94)	119.89	<0.00001	95%	0.03
Long travel time/distance to the first healthcare provider	11	1.39 (1.08, 1.78)	36.06	<0.0001	72%	0.01
Age (older)	31	1.01 (0.91, 1.11)	144.10	<0.00001	79%	0.87
Education(illiterate)	14	0.99 (0.78, 1.24)	45.23	<0.0001	71%	0.90
Rural residence	14	1.15 (0.99, 1.34)	42.25	<0.0001	69%	0.06
Chest radiographs	3	1.42 (0.78, 2.58)	27.72	<0.0001	93%	0.25
Consult with a public hospital	10	0.99 (0.65, 1.50)	66.39	<0.00001	86%	0.96

a. P-value for homogeneous studies (Q statistic, P≥0.10, or P<0.10, but I^2^≤50%)—fixed-effect model; otherwise, P-value for significantly heterogeneous studies (Q statistic, P<0.10, but I^2^>50%)—random effects model.

### Provider delay studies

The provider (usually described as the health care system, doctor, or diagnostician) delay studies all used the first consultation at a health facility as the starting point. However, 18 studies used diagnosis as the endpoint, whereas 12 others used the start of anti-TB treatment. Moreover, different endpoint definitions may not necessarily be different points in time. The duration was ranged from 7 to 79 days.

#### Factors assessed

There were 30 studies associated with provider delay. The associated factors may be related to the aspect of provider delay for diagnosis and treatment. Among socio-demographic factors, 24 studies mentioned a connection between male sex and provider delay: two studies showed a positive relationship, two showed a negative relationship, and 20 showed no relationship. Other factors included older age (k = 24), illiterate or lack of education (k = 10), rural residence (k = 8), low income (k = 10), and unemployed (k = 4). Other factors, such as symptoms (cough, k = 2; hemoptysis, k = 8), examination (positive sputum smears: k = 8; chest radiographs: k = 5), and consulting with a public hospital (k = 12), were found to be correlated with provider delay in at least one study. Finally long travel time or distance from the patient’s home to the health facility was reported to be correlated with provider delay in seven studies. (Table 5)

**Table 5:** Factors assessed for association with provider delay (k = 30).

Citation	Delay = 1st sx to (interval)	Male sex	Old age	Education	Unemployed	Rural residence	Income low level	Haemoptysis	Cough	Smear positive	Chest radiographs	Consult with a public hospital	Long travel time/distance
Wang W (2007) [18]	Diagnosis (8 days)	x	x	x	x	—	x	x	—	—	x	—	—
Yamasaki-Nakagawa M (2001) [22]	Diagnosis (1 month)	—	—	—	—	—	—	—	—	—	—	—	—
Rojpibulstit M (2006) [24]	Diagnosis (interval ot recorded)	x	↑	—	—	—	—	—	—	x	—	↑	—
Zhou C (2012) [27]	Diagnosis (14 days)	x	↓	x	—	x	x	—	—	—	—	↓	↑
Chang CT (2007) [28]	Diagnosis (22 days)	x	—	—	—	—	x	x	—	—	—	—	—
Basnet R (2009) [30]	Diagnosis (30 days)	x	x	x	—	—	x	—	—	x	—	—	x
Xu X (2013) [36]	Diagnosis (2 weeks)	x	x	—	—	—	—	—	—	↓	—	—	—
Cheng G (2005) [39]	Diagnosis (interval not recorded)	↑	x	x	—	—	x	—	—	—	—	x	x
Shu W (2014) [42]	Diagnosis (interval not recorded)	—	—	—	—	—	—	—	—	↓	—	—	—
Ngamvithayapong J (2001) [43]	Diagnosis (7 days)	↓	x	x	—	—	—	—	—	—	—	—	—
Kelkar-Khambete A (2008) [46]	Diagnosis (7 days)	x	x	x	—	x	↑	—	—	—	—	—	—
Bai LQ (2004) [53]	Diagnosis (14 days)	—	—	—	—	—	—	x	—	—	—	—	—
Sabawoon W (2012) [55]	Diagnosis (interval not recorded)	—	—	—	—	—	—	—	—	—	—	—	↑
Karim F (2007) [56]	Diagnosis (interval not recorded)	x	x	—	—	—	—	—	—	—	—	—	—
Lönnroth K (1999) [58]	Diagnosis (4 weeks)	↓	—	—	—	↓	—	↓	—	—	—	↑	—
Rajeswari R (2002) [59]	Diagnosis (7days)	x	↑	x	↑	x	x	—	—	—	—	x	↑
Lin HP (2009) [60]	Diagnosis (9 days)	x	↑	—	—	—	—	—	—	↓	Aberrant: ↓	—	—
Wang WB (2006) [61]	Diagnosis (interval not recorded)	x	x	x	—	—	x	↑	—	—	x	—	—
Leung EC (2007) [19]	Anti-TB treatment (20 days)	—	↑	—	—	—	—	—	—	—	↓	—	—
Huong NT (2007) [20]	Anti-TB treatment (6 weeks)	x	x	—	—	↓	—	—	—	—	—	↑	—
Enkhbat S (1996) [21]	Anti-TB treatment (1 month)	x	x	—	—	x	x	—	—	—	—	x	—
Chiang CY (2005) [23]	Anti-TB treatment (23 days)	↑	x	—	—	—	—	↑	↓	↑	↑	↑	—
Selvam JM (2003) [44]	Anti-TB treatment (14 days)	x	x	↑	x	—	—	—	—	—	—	↓	↑
Ayé R (2010) [47]	Anti-TB treatment (interval not recorded)	x	x	—	—	x	—	—	—	—	—	x	—
Tobgay KJ (2006) [48]	Anti-TB treatment (7 days)	x	x	—	—	—	—	—	—	—	—	↓	—
Mor Z (2013) [51]	Anti-TB treatment (79 days)	x	x	—	x	—	—	—	—	x	—	—	—
Rifat M (2011) [52]	Anti-TB treatment (9 weeks)	x	↑	—	—	↑	—	—	—	—	—	—	—
Tamhane A (2012) [54]	Anti-TB treatment (14 days)	—	x	x	—	—	x	x	—	—	—	—	x
Thakur R (2010) [57]	Anti-TB treatment (1 day)	x	x	x	—	x	x	—	—	—	—	↓	—
Lin CY (2010) [62]	Anti-TB treatment (7 days)	x	↑	—	—	—	—	x	x	↓	—	—	—

x. Assessed but P ≥ 0.05

↓. Negatively correlated with delay, P < 0.05

↑. Positively correlated with delay, P < 0.05

—. Not assessed.

#### Meta-analysis

**Fig 3:**
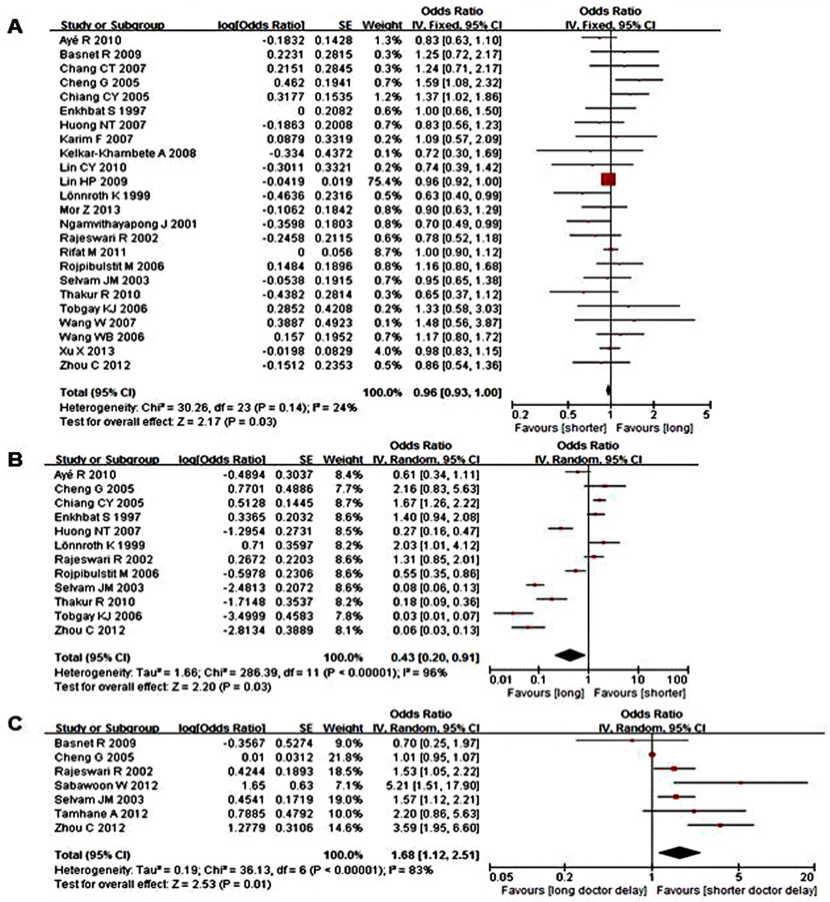
Forest plots of meta-analysis of factors associated with provider delay. This figure shows forest plots for the meta-analysis of male sex factor associated with provider delay. OR and 95% CI for the factor are given. Notes: A- Male sex; B- Consult with a public hospital; C- Long travel time/distance to the first healthcare provider.

Male sex, older age, illiterate or lack of education, unemployment, rural residence, low income, hemoptysis, positive sputum smears, consulting with a public hospital, and long travel time or distance had sufficient information to calculate ORs and 95% CIs. Only male sex and consultation with a public hospital negatively correlated with provider delays, whereas long travel time or distance to the first healthcare provider positively associated with provider delay (P<0.05). The pooled effect size for the factors in these studies are shown in Table 6, indicating that male patients and the first consultation with a public hospital had shorter provider delays, whereas long travel time or distance to the first healthcare provider may have longer delays (Fig. 3).

**Table 6:** Results of meta-analyses and test for heterogeneity of provider delay.

Variable	k	OR (95% CI) ^a^	Heterogeneity between studies			Test for overall effect (P)
			Q statistic	P-value	I^2^	
Male sex	24	0.96 (0.93, 1.00)	30.26	0.14	24%	0.03
Consult with a public hospital	12	0.43 (0.20, 0.91)	286.39	<0.00001	96%	0.03
Long travel time/distance to the first healthcare provider	7	1.68 (1.12, 2.51)	36.13	<0.00001	83%	0.01
Age (older)	24	1.17 (0.91, 1.51)	962.61	<0.00001	98%	0.21
Education (illiterate)	10	0.88 (0.74, 1.05)	7.32	0.60	0%	0.15
Rural residence	8	1.20 (0.76, 1.88)	62.37	<0.00001	89%	0.44
Income (low)	10	1.13 (0.80, 1.58)	27.44	0.0010	67%	0.50
Unemployed	4	1.68 (0.72, 3.94)	38.51	<0.00001	92%	0.23
Haemoptysis	8	0.78 (0.42, 1.44)	53.68	<0.00001	87%	0.42
Positive smear	8	0.61 (0.13, 2.96)	2313.32	<0.00001	100%	0.54
Chest radiographs	5	0.72 (0.31, 1.70)	182.38	<0.00001	98%	0.46

a. P-value for homogeneous studies (Q statistic, P≥0.10, or P<0.10, but I^2^≤50%)—fixed-effect model; otherwise, P-value for significantly heterogeneous studies (Q statistic, P<0.10, but I^2^>50%)—random effects model.

## Discussion

Although the prevalence of TB in most Asian countries has been decreasing annually in recent years [63], there is still an epidemiological challenge for TB control. Moreover, case detection is often late. This meta-analysis reviewed the factors correlated to delays in TB diagnosis and treatment, which could provide valuable data for TB programs. A meta-analysis regarding TB treatment delays in Asia was not previously conducted. Understanding the factors that are closely associated with delays is essential to effectively controlling TB, accessing TB services, as well as reducing delays for seeking prompt diagnosis and treatment [64]. As reported by Glanz et al. [65], individual-level factors include socio-demographics, TB symptoms, knowledge, attitudes, and behaviors. Furthermore, due to the ease of data collection, socio-demographic characteristics were the majority of independent variables that were assessed for correlation with patient or provider delays.

Our data showed that male sex served as an important determinant of both patient and provider delays. Despite the cross-sectional nature and sufficient sample size to detect correlations with delay, male sex negatively correlated with delays in seeking diagnosis and treatment for TB. Similar findings were reported in studies from other countries [66, 67]. In addition, cultural factors are likely to be involved in gender differences. Because of the higher TB prevalence in males, TB is likely suspected and investigated more readily in men [68]. Therefore, men are likely to seek health care earlier when symptoms appeared; thus, TB propaganda regarding disease control and diagnosis is more necessary for men. Moreover, biological differences between males and females with respect to TB incidence and the sensitivity of smear examinations may play an essential role [69]. In addition, gender-specific themes were particularly pervasive within financial, stigma, and health literacy barriers. Studies [70, 71] for gender-related differences in barriers and delays in TB diagnosis and treatment services found that women experienced greater financial barriers and greater stigma than men; the identified barriers were gender-specific in the majority of studies. It is therefore challenging to overcome gender-specific barriers that require intervention. We propose that female patients with symptoms suggestive of TB should receive more attention before and after seeking care.

Regarding the behavior of patients, long travel time or distance to the first healthcare provider was found to be a significant determinant that correlated with both patient and provider delays. Previous reviews of the factors associated with patient and diagnostic delays in China and sub-Saharan African countries [14, 15] reported that the majority of factors associated with delays in TB diagnosis and treatment were related to the place of first consultation, travel time, or distance from the health facility. A study conducted in rural Ethiopia agreed that the difficulty of geographic access to health facilities was identified as one of the primary reasons for patient delay [72], indicating that geographic issues can also influence the timeliness of medical service, particularly in remote and poor areas.

This meta-analysis highlights that socio-demographic factors, including low level of income and unemployment, were positively associated with long patient delay. According to WHO determinants of TB control, risk factors of delayed diagnosis can be divided into three levels: socio-economic, clinical, and health system factors [73]. Most Asian countries have a considerable rural population due to their developing economics. Due to lower incomes, potential TB patients may be less likely to seek care after persistent cough for 3 weeks compared to higher-income individuals [74]. Additionally, there are fewer health facilities in low-economic areas and it is more difficult for patients to seek health-care, subsequently affecting patient delay. Previous studies have shown that unemployment is an independent risk factor for delay in seeking treatment [58]. Pooling the factors together, the data indicate that poverty may discourage patients from seeking health care in a timely manner. Community health workers should design individualized and community-based propaganda and education to address the symptoms of TB. Moreover, community health workers can be effective in the referral of potential patients to rural and national TB control institutions. Therefore, when there is a global emphasis on the use of community health service personnel for disease prevention and control at the rural level, rural health workers could play a key role in the fight against TB [75].

Another factor associated with patient delay was hemoptysis, which negatively correlated with delay. The presence of hemoptysis may be the most characteristic and severe symptom of TB, and it is not surprising that it prompts the initial health consultation [25]. A previous study suggested why hemoptysis can lead to timely physical examinations and a TB diagnosis [76]. It is important to balance the TB severity and the costs associated with seeking care to continue with diagnosis and treatment. In the current report, consultation with a public hospital for the first visit negatively correlated with provider delays. Storla et al. [77] reported similar results in a systematic review, showing that selection of a traditional practitioner for the first visit is associated with prolonged delays in diagnosis in a range of studies. It was also reported that the number and type of provider first consulted were the most important risk factors for diagnosis delay [13]. Thus, understanding how patients seek health care and to attempt to provide sufficient public health care is very important for TB control.

This meta-analysis was limited by the study designs of the included articles, which may explain the heterogeneity to some extent. Almost all included studies were cross-sectional in design, which used a consecutive method of sampling. Cross-sectional studies have a number of inherent limitations that potentially bias the results, such as its low comparability and that it is includes certain confounders. Thus, these studies do not obtain the causality of the observed associations. Although some of the studies were controlled by logistic regressions for these confounders, their residual effect might still have introduced bias. In addition, although the NOS scores of included studies were a little low, they were still in the moderate quality (most 5 or 6 scores) [78,79]. Therefore, it should make the the reliability of this review.

The available studies had different definitions of provider delay, as well as multiple classifications of socio-demographic variables, such as income, education, and types of health facility consulted by TB patients, which limit the comparability of these studies. Although there were varying cut-off points for delay duration (particularly for provider delay), most studies used the same starting point and end point (provider delay had two different end points). However, when the two types of delay are combined, the correlated factors of delays can be better analyzed [24].

## Conclusion

This meta-analysis confirms that socio-demographic characteristics, symptoms of TB patients, and long travel distance to the first provider have critical roles in patient delay in TB diagnosis and treatment. However, only male sex and long travel distance to the first provider correlated to both patient and provider delays, suggesting that availability, and particularly timeliness, of public healthcare providers should be primary concerns of policymakers. However, these studies are not comparable because of their weak and sole study designs and various cut-off points for the duration of delays, particularly for provider delay. Future studies are required that use stronger designs (cohort or case-control studies) and can establish causality among affected factors. Moreover, a standard definition of delay will allow for more appropriate comparisons across studies.

## Supporting Information

S1 Table PRISMA Checklist. (DOC)

S2 Table List of Reasons for Exclusion 42 Full-text Articles. (DOC)

S3 Table Results of subgroup analysis and test for heterogeneity of provider’s delay. (DOC)

S4 Table List of all the scores for each item of the NOS of the included 45 studies. (DOC)

## References

[1] Goodchild M, Sahu S, Wares F, Dewan P, Shukla RS, Chauhan LS, et al A cost-benefit analysis of scaling up tuberculosis control in India. Int J Tuberc Lung Dis. 2011;15(3): 358–362. 21333103

[2] WHO. China: health, poverty and economic development. 2005 Dec [cited 17 August 2014] Geneva: World Health Organization Available: www.who.int/macrohealth/action/CMH_China.pdf.

[3] WHO. Global Tuberculosis Report 2013. 2013 Nov [cited 17 August 2014] Geneva: World Health Organization Available: http://apps.who.int/iris/bitstream/10665/91355/1/9789241564656_eng.pdf.

[4] WHO. Fact sheets on tuberculosis. 2013 [cited 17 August 2014] Geneva: World Health Organization Available: http://www.who.int/tb/publications/factsheet_global.pdf?ua=1.

[5] Bjune G. Tuberculosis in the 21st century: an emerging pandemic? Nor Epidemiol. 2005;15(2): 133–139.

[6] Yimer S, Bjune G, Alene G. Diagnostic and treatment delay among pulmonary tuberculosis patients in Ethiopia: a cross sectional study. BMC Infect Dis. 2005;5: 112 16343350 10.1186/1471-2334-5-112 PMC1326202

[7] Ward HA, Marciniuk DD, Pahwa P, Hoeppner VH. Extent of pulmonary tuberculosis in patients diagnosed by active compared to passive case finding. Int J Tuberc Lung Dis. 20048(5): 593–597. 15137536

[8] Aldhubhani AH, lzham MI, Pazilah I, Anaam MS. Effect of delay in diagnosis on the rate of tuberculosis among close contacts of tuberculosis patients. East Mediterr Health J. 2013;19(10): 837–842. 24313146

[9] Nkosi D, Janssen S, Padanilam X, Louw R, Menezes CN, Grobusch MP. Factors influencing specialist care referral of multidrug- and extensively drug-resistant tuberculosis patients in Gauteng/South Africa: a descriptive questionnaire-based study. BMC Health Serv Res. 2013;13: 268 10.1186/1472-6963-13-268 23837531 PMC3708743

[10] Farah MG, Rygh JH, Steen TW, Selmer R, Heldal E, Bjune G. Patient and health care system delays in the start of tuberculosis treatment in Norway. BMC Infect Dis. 2006;6: 33 16504113 10.1186/1471-2334-6-33 PMC1435913

[11] WHO. Tuberculosis in the WHO South-East Asia Region. 2010 [cited 17 August 2014] Geneva: World Health Organization Available: http://www.who.int/bulletin/volumes/88/3/09-073874/en/. 10.2471/BLT.09.073874 PMC2828794 20428378

[12] Sreeramareddy CT, Panduru KV, Menten J, Van den Ende J. Time delays in diagnosis of pulmonary tuberculosis: a systematic review of literature. BMC Infect Dis. 2009;9: 91 10.1186/1471-2334-9-91 19519917 PMC2702369

[13] Sreeramareddy CT, Qin ZZ, Satyanarayana S, Subbaraman R, Pai M. Delays in diagnosis and treatment of pulmonary tuberculosis in India: a systematic review. Int J Tuberc Lung Dis. 2014;18(3): 255–266. 10.5588/ijtld.13.0585 24670558 PMC4070850

[14] Li Y, Ehiri J, Tang S, Li D, Bian Y, Lin H, et al Factors associated with patient, and diagnostic delays in Chinese TB patients: a systematic review and meta-analysis. BMC Med. 2013;11: 156 10.1186/1741-7015-11-156 23819847 PMC3699418

[15] Finnie RK, Khoza LB, van den Borne B, Mabunda T, Abotchie P, Mullen PD. Factors associated with patient and health care system delay in diagnosis and treatment for TB in sub-Saharan African countries with high burdens of TB and HIV. Trop Med Int Health. 2011;16(4): 394–411. 10.1111/j.1365-3156.2010.02718.x 21320240

[16] Glynn JR. Resurgence of tuberculosis and the impact of HIV infection. Br Med Bull. 1998;54(3): 579–593. 10326286 10.1093/oxfordjournals.bmb.a011712

[17] Wells GA, Shea B, O’connell D, Peterson JEA, Welch V, Losos M, et al. The Newcastle-Ottawa Scale (NOS) for assessing the quality of nonrandomised studies in meta-analyses. 2000. Available: http://www.medicine.mcgill.ca/rtamblyn/Readings/The%20Newcastle%20-%20Scale%20for%20assessing%20the%20quality%20of%20nonrandomised%20studies%20in%20meta-analyses.pdf.

[18] Wang W, Jiang Q, Abdullah AS, Xu B. Barriers in accessing to tuberculosis care among non-residents in Shanghai: a descriptive study of delays in diagnosis. Eur J Public Health. 2007;17(5): 419–423. 17412714 10.1093/eurpub/ckm029

[19] Leung EC, Leung CC, Tam CM. Delayed presentation and treatment of newly diagnosed pulmonary tuberculosis patients in Hong Kong. Hong Kong Med J. 2007;13(3): 221–227. 17548911

[20] Huong NT, Vree M, Duong BD, Khanh VT, Loan VT, Co NV, et al Delays in the diagnosis and treatment of tuberculosis patients in Vietnam: a cross-sectional study. BMC Public Health. 2007;7: 110 17567521 10.1186/1471-2458-7-110 PMC1906755

[21] Enkhbat S, Toyota M, Yasuda N, Ohara H. Differing influence on delays in the case-finding process for tuberculosis between general physicians and specialists in Mongolia. J Epidemiol. 1997;7(2): 93–98. 9255030 10.2188/jea.7.93

[22] Yamasaki-Nakagawa M, Ozasa K, Yamada N, Osuga K, Shimouchi A, Ishikawa N, et al Gender difference in delays to diagnosis and health care seeking behaviour in a rural area of Nepal. Int J Tuberc Lung Dis. 2001;5(1): 24–31. 11263512

[23] Chiang CY, Chang CT, Chang RE, Li CT, Huang RM. Patient and health system delays in the diagnosis and treatment of tuberculosis in Southern Taiwan. Int J Tuberc Lung Dis. 2005;9(9): 1006–1012. 16158893

[24] Rojpibulstit M, Kanjanakiritamrong J, Chongsuvivatwong V. Patient and health system delays in the diagnosis of tuberculosis in Southern Thailand after health care reform. Int J Tuberc Lung Dis. 2006;10(4): 422–428. 16602407

[25] Li X, Jiang S, Li X, Mei J, Zhong Q, Xu W, et al Predictors on delay of initial health-seeking in new pulmonary tuberculosis cases among migrants population in East China. PLoS One. 2012;7(2): e31995 10.1371/journal.pone.0031995 22384123 PMC3285186

[26] Lock WA, Ahmad RA, Ruiter RA, van der Werf MJ, Bos AE, Mahendradhata Y, et al Patient delay determinants for patients with suspected tuberculosis in Yogyakarta province, Indonesia. Trop Med Int Health. 2011;16(12): 1501–1510. 10.1111/j.1365-3156.2011.02864.x 21838716

[27] Zhou C, Tobe RG, Chu J, Gen H, Wang X, Xu L. Detection delay of pulmonary tuberculosis patients among migrants in China: a cross-sectional study. Int J Tuberc Lung Dis. 2012;16(12): 1630–1636. 10.5588/ijtld.12.0227 23131261

[28] Chang CT, Esterman A. Diagnostic delay among pulmonary tuberculosis patients in Sarawak, Malaysia: a cross-sectional study. Rural Remote Health. 2007;7(2): 667 17511524

[29] Lin X, Chongsuvivatwong V, Geater A, Lijuan R. The effect of geographical distance on TB patient delays in a mountainous province of China. Int J Tuberc Lung Dis. 2008;12(3): 288–293. 18284834

[30] Basnet R, Hinderaker SG, Enarson D, Malla P, Mørkve O. Delay in the diagnosis of tuberculosis in Nepal. BMC Public Health. 2009;9: 236 10.1186/1471-2458-9-236 19602255 PMC2716339

[31] Güneylioglu D, Yilmaz A, Bilgin S, Bayram U, Akkaya E. Factors affecting delays in diagnosis and treatment of pulmonary tuberculosis in a tertiary care hospital in Istanbul, Turkey. Med Sci Monit. 2004;10(2): CR62–67. 14737045

[32] Hoa NB, Tiemersma EW, Sy DN, Nhung NV, Vree M, Borgdorff MW, et al Health-seeking behaviour among adults with prolonged cough in Vietnam. Trop Med Int Health. 2011;16(10):1260–1267. 10.1111/j.1365-3156.2011.02823.x 21692960

[33] Xu B, Diwan VK, Bogg L. Access to tuberculosis care: what did chronic cough patients experience in the way of healthcare-seeking? Scand J Public Health. 2007;35(4): 396–402. 17786803 10.1080/14034940601160664

[34] Ahmad RA, Richardus JH, de Vlas SJ. Care-seeking behaviour among individuals with TB symptoms in Jogjakarta Province, Indonesia: a community-based study. Int Health. 2013 3;5(1):51–7. 10.1093/inthealth/ihs002 24029846

[35] Phoa LL, Teleman MD, Wang YT, Chee CB. Characteristics of patients with delayed diagnosis of infectious pulmonary tuberculosis. Respirology. 2005;10(2): 196–200. 15823185 10.1111/j.1440-1843.2005.00644.x

[36] Xu X, Liu JH, Cao SY, Zhao Y, Dong XX, Liang Y, et al Delays in care seeking, diagnosis and treatment among pulmonary tuberculosis patients in Shenzhen, China. Int J Tuberc Lung Dis. 2013;17(5): 615–620. 10.5588/ijtld.12.0231 23575326

[37] Rundi C, Fielding K, Godfrey-Faussett P, Rodrigues LC, Mangtani P. Delays in seeking treatment for symptomatic tuberculosis in Sabah, East Malaysia: factors for patient delay. Int J Tuberc Lung Dis. 2011;15(9): 1231–1238, 10.5588/ijtld.10.0585 21943851

[38] Zhao X, Yang P, Gai R, Mei L, Wang X, Xu L. Determinants of health care-seeking delay among tuberculosis patients in Shandong Province, China. Eur J Public Health. 2014;24(5): 757–761. 10.1093/eurpub/ckt113 23975893

[39] Cheng G, Tolhurst R, Li RZ, Meng QY, Tang S. Factors affecting delays in tuberculosis diagnosis in rural China: a case study in four counties in Shandong Province. Trans R Soc Trop Med Hyg. 2005;99(5): 355–362. 15780342 10.1016/j.trstmh.2004.07.005

[40] Tobe RG, Xu L, Zhou C, Yuan Q, Geng H, Wang X. Factors affecting patient delay of diagnosis and completion of Direct Observation Therapy, Short-course (DOTS) among the migrant population in Shandong, China. Biosci Trends. 2013;7(3): 122–128. 23836035

[41] Choudhari M, Jha N, Yadav DK, Chaudhary D. Factors associated with patient delay in diagnosis of pulmonary tuberculosis in a district. J Nepal Health Res Counc. 2012;10(22): 234–238. 23281458

[42] Shu W, Chen W, Zhu S, Hou Y, Mei J, Bai L, et al Factors causing delay of access to tuberculosis diagnosis among new, active tuberculosis patients: a prospective cohort study. Asia Pac J Public Health. 2014;26(1):33–41. 10.1177/1010539513502523 24097935

[43] Ngamvithayapong J, Yanai H, Winkvist A, Diwan V. Health seeking behaviour and diagnosis for pulmonary tuberculosis in an HIV-epidemic mountainous area of Thailand. Int J Tuberc Lung Dis. 2001;5(11): 1013–1020. 11716337

[44] Selvam JM, Wares F, Perumal M, Gopi PG, Sudha G, Chandrasekaran V, et al Health-seeking behaviour of new smear-positive TB patients under a DOTS programme in Tamil Nadu, India, 2003. Int J Tuberc Lung Dis. 2007;11(2): 161–167. 17263286

[45] Chen HG, Liu M, Jiang SW, Gu FH, Huang SP, Gao TJ, et al Impact of diabetes on diagnostic delay for pulmonary tuberculosis in Beijing. Int J Tuberc Lung Dis. 2014;18(3): 267–271. 10.5588/ijtld.13.0140 24670559

[46] Kelkar-Khambete A, Kielmann K, Pawar S, Porter J, Inamdar V, Datye A, et al India's Revised National Tuberculosis Control Programme: looking beyond detection and cure. Int J Tuberc Lung Dis. 2008;12(1): 87–92. 18173883

[47] Ayé R, Wyss K, Abdualimova H, Saidaliev S. Patient's site of first access to health system influences length of delay for tuberculosis treatment in Tajikistan. BMC Health Serv Res. 2010;10: 10 10.1186/1472-6963-10-10 20064224 PMC2822832

[48] Tobgay KJ, Sarma PS, Thankappan KR. Predictors of treatment delays for tuberculosis in Sikkim. Natl Med J India. 2006;19(2): 60–63. 16756190

[49] Rumman KA, Sabra NA, Bakri F, Seita A, Bassili A. Prevalence of tuberculosis suspects and their healthcare-seeking behavior in urban and rural Jordan. Am J Trop Med Hyg. 2008;79(4): 545–551. 18840742

[50] Wang Y, Long Q, Liu Q, Tolhurst R, Tang S. Treatment seeking for symptoms suggestive of TB: comparison between migrants and permanent urban residents in Chongqing, China. Trop Med Int Health. 2008;13(7): 927–933. 10.1111/j.1365-3156.2008.02093.x 18482198

[51] Mor Z, Kolb H, Lidji M, Migliori G, Leventhal A. Tuberculosis diagnostic delay and therapy outcomes of non-national migrants in Tel Aviv, 1998–2008. Euro Surveill. 2013;18(12): pii 20433. 23557947

[52] Rifat M, Rusen ID, Islam MA, Enarson DA, Ahmed F, Ahmed SM, et al Why are tuberculosis patients not treated earlier? A study of informal health practitioners in Bangladesh. Int J Tuberc Lung Dis. 2011;15(5): 647–651. 10.5588/ijtld.10.0205 21756516

[53] Bai LQ, Xiao SY. Factors associated with diagnostic delay for patients with smear-positive pulmonary tuberculosis in rural Hunan, China. Zhonghua Jie He He Hu Xi Za Zhi. 2004;27(9): 617–620. 15498275

[54] Tamhane A, Ambe G, Vermund SH, Kohler CL, Karande A, Sathiakumar N. Pulmonary tuberculosis in mumbai, India: factors responsible for patient and treatment delays. Int J Prev Med. 2012;3(8): 569–580. 22973488 PMC3429805

[55] Sabawoon W, Sato H, Kobayashi Y. Delay in the treatment of pulmonary tuberculosis: a report from Afghanistan. Environ Health Prev Med. 2012;17(1): 53–61. 10.1007/s12199-011-0219-9 21590428 PMC3258319

[56] Karim F, Islam MA, Chowdhury AM, Johansson E, Diwan VK. Gender differences in delays in diagnosis and treatment of tuberculosis. Health Policy Plan. 2007;22(5): 329–334. 17698889 10.1093/heapol/czm026

[57] Thakur R, Murhekar M. Delay in diagnosis and treatment among TB patients registered under RNTCP Mandi, Himachal Pradesh, India, 2010. Indian J Tuberc. 2013;60(1): 37–45. 23540087

[58] Lönnroth K, Thuong LM, Linh PD, Diwan VK. Delay and discontinuity—a survey of TB patients' search of a diagnosis in a diversified health care system. Int J Tuberc Lung Dis. 1999;3(11): 992–1000. 10587321

[59] Rajeswari R, Chandrasekaran V, Suhadev M, Sivasubramaniam S, Sudha G, Renu G. Factors associated with patient and health system delays in the diagnosis of tuberculosis in South India. Int J Tuberc Lung Dis. 2002;6(9): 789–795. 12234134

[60] Lin HP, Deng CY, Chou P. Diagnosis and treatment delay among pulmonary tuberculosis patients identified using the Taiwan reporting enquiry system, 2002–2006. BMC Public Health. 2009;9: 55 10.1186/1471-2458-9-55 19216733 PMC2654887

[61] Wang WB, Xiu Y, Jiang WL, Jiang QW, Xu B. Diagnostic delay and its factors in access to tuberculosis care in northern rural Jiangsu province. Fudan Xuebao (Yixuekexueban). 2006; 33(1): 33–38.

[62] Lin CY, Lin WR, Chen TC, Lu PL, Huang PM, Tsai ZR, et al Why is in-hospital diagnosis of pulmonary tuberculosis delayed in southern Taiwan? J Formos Med Assoc. 2010;109(4): 269–277. 10.1016/S0929-6646(10)60052-6 20434036

[63] WHO. Cases: Mortality and prevalence by country (all years). 2007 [cited 17 August 2014] Geneva: World Health Organization Available: http://apps.who.int/gho/data/view.main.57020ALL?lang=en

[64] WHO. Early detection of tuberculosis: an overview of approaches, guidelines and tools. 2011 [cited 17 August 2014] Geneva: World Health Organization Available: https://extranet.who.int/iris/restricted/bitstream/10665/70824/1/WHO_HTM_STB_PSI_2011.21_eng.pdf.

[65] Glanz KR, Rimer BK, Viswanath K. Health behavior and health education: Theory, research, and practice. 4th ed. John Wiley & Sons; 2008.

[66] Sendagire I, Schim Van der Loeff M, Mubiru M, Konde-Lule J, Cobelens F. Long delays and missed opportunities in diagnosing smear-positive pulmonary tuberculosis in Kampala, Uganda: a cross-sectional study. PLoS One. 2010;5(12): e14459 10.1371/journal.pone.0014459 21206746 PMC3012078

[67] Jurcev-Savicevic A, Mulic R, Kozul K, Ban B, Valic J, Bacun-Ivcek L, et al Health system delay in pulmonary tuberculosis treatment in a country with an intermediate burden of tuberculosis: a cross-sectional study. BMC Public Health. 2013;13: 250 10.1186/1471-2458-13-250 23517315 PMC3608169

[68] Jurcev-Savicevic A, Kardum G. Health-care seeking behaviour for tuberculosis symptoms in Croatia. Eur J Public Health. 2012;22(4): 573–577. 10.1093/eurpub/ckr132 21920849

[69] Long NH, Diwan VK, Winkvist A. Difference in symptoms suggesting pulmonary tuberculosis among men and women. J Clin Epidemiol. 2002;55(2): 115–120. 11809348 10.1016/s0895-4356(01)00455-3

[70] Yang WT, Gounder CR, Akande T, De Neve JW, McIntire KN, Chandrasekhar A, et al Barriers and delays in tuberculosis diagnosis and treatment services: does gender matter? Tuberc Res Treat. 2014;2014: 461935 10.1155/2014/461935 24876956 PMC4020203

[71] Krishnan L, Akande T, Shankar AV, McIntire KN, Gounder CR, Gupta A, et al Gender-related barriers and delays in accessing tuberculosis diagnostic and treatment services: a systematic r1eview of qualitative studies. Tuberc Res Treat. 2014;2014: 215059 10.1155/2014/215059 24900921 PMC4037602

[72] Tadesse T, Demissie M, Berhane Y, Kebede Y, Abebe M. Long distance travelling and financial burdens discourage tuberculosis DOTs treatment initiation and compliance in Ethiopia: a qualitative study. BMC Public Health. 2013;13: 424 10.1186/1471-2458-13-424 23634650 PMC3644232

[73] WHO. The global plan to stop TB 2011–2015: transforming the fight towards elimination of tuberculosis. 2010 [cited 17 August 2014] Geneva: World Health Organization Available: http://apps.who.int/iris/bitstream/10665/44437/1/9789241500340_eng.pdf?ua=1. 10.1371/journal.pone.0108591

[74] Zhang T, Tang S, Jun G, Whitehead M. Persistent problems of access to appropriate, affordable TB services in rural China: experiences of different socio-economic groups. BMC Public Health. 2007;7: 19 17288593 10.1186/1471-2458-7-19 PMC1805429

[75] Hipgrave D. Communicable disease control in China: From Mao to now. J Glob Health. 2011;1(2): 224–238. 23198121 PMC3484775

[76] Meyssonnier V, Li X, Shen X, Wang H, Li DY, Liu ZM, et al Factors associated with delayed tuberculosis diagnosis in China. Eur J Public Health. 2013;23(2): 253–257. 10.1093/eurpub/cks037 22874738

[77] Storla DG, Yimer S, Bjune GA. A systematic review of delay in the diagnosis and treatment of tuberculosis. BMC Public Health. 2008;8: 15 10.1186/1471-2458-8-15 18194573 PMC2265684

[78] van Dijk GM, Maneva M, Colpani V, Dhana K, Muka T, Jaspers L, et al The association between vasomotor symptoms and metabolic health in peri- and postmenopausal women: A systematic review. Maturitas. 2015;80(2): 140–147. 10.1016/j.maturitas.2014.11.016 25532993

[79] Bechard LJ, Rothpletz-Puglia P, Touger-Decker R, Duggan C, Mehta NM. Influence of obesity on clinical outcomes in hospitalized children: a systematic review. JAMA Pediatr. 2013;167(5): 476–482. 10.1001/jamapediatrics.2013.13 23478891 PMC4743026

