# Supplementary material for: Factors Associated with Patient and Provider Delays for Tuberculosis Diagnosis and Treatment in Asia: A Systematic Review and Meta-Analysis
Source: PLoS One. 2015 Mar 25;10(3):e0120088. doi: 10.1371/journal.pone.0120088 (PMC4373856; doi:10.1371/journal.pone.0120088)
Supplement: S2 Table — (DOC) [file pone.0120088.s002.doc]

**S2 Table - List of Reasons for Exclusion 42 Full-text Articles**

| **No.** | **Exclusion articles** | **Reasons** |
| --- | --- | --- |
| 1 | Mori T, Shimao T, Jin BW, Kim SJ. Analysis of case-finding process of tuberculosis in Korea. Tuber Lung Dis. 1992 Aug;73(4):225-31. | Only description of delay, not about the factors association with delay |
| 2 | Long Q, Li Y, Wang Y, Yue Y, Tang C, Tang S, Squire SB, Tolhurst R. Barriers to accessing TB diagnosis for rural-to-urban migrants with chronic cough in Chongqing, China: a mixed methods study. BMC Health Serv Res. 2008 Oct 2;8:202. doi: 10.1186/1472-6963-8-202. | Only description of delay, not about the factors association with delay |
| 3 | Charles N, Thomas B, Watson B, Raja Sakthivel M, Chandrasekeran V, Wares F. Care seeking behavior of chest symptomatics: a community based study done in South India after the implementation of the RNTCP. PLoS One. 2010 Sep 20;5(9). pii: e12379. doi: 10.1371/journal.pone.0012379. | Only description of delay, not about the factors association with delay |
| 4 | Hooi LN. Case-finding for pulmonary tuberculosis in Penang. Med J Malaysia. 1994 Sep;49(3):223-30. | Only description of delay, not about the factors association with delay |
| 5 | Mesfin MM, Newell JN, Madeley RJ, Mirzoev TN, Tareke IG, Kifle YT, Gessessew A, Walley JD. Cost implications of delays to tuberculosis diagnosis among pulmonary tuberculosis patients in Ethiopia. BMC Public Health. 2010 Mar 30;10:173. doi: 10.1186/1471-2458-10-173. | Only description of delay, not about the factors association with delay |
| 6 | Lee Chih-Hsin, Wang Jann-Yuan, Chen Hsin-YiChang Hou-Tai. (2013) Delay in diagnosis of pulmonary tuberculosis and risk of nosocomial transmission among intensive care units: a national surveillance in taiwan.Respirology 18: 10-10. | Only description of delay, not about the factors association with delay |
| 7 | Charoenmak B, Worayuttakarn S, Chusri S, Silpapojakul K. Delay in pulmonary tuberculosis suspicion and isolation among hospitalized patients: Songklanagarind Hospital perspective. J Med Assoc Thai. 2012 Apr;95(4):493-6. | Only description of delay, not about the factors association with delay |
| 8 | Liam CK, Tang BG. Delay in the diagnosis and treatment of pulmonary tuberculosis in patients attending a university teaching hospital. Int J Tuberc Lung Dis. 1997 Aug;1(4):326-32. | Only description of delay, not about the factors association with delay |
| 9 | Okutan O, Kartaloglu Z, Cerrahoglu K, Ilvan A, Tozkoparan E, Aydilek R. Delay in the diagnosis of Turkish servicemen with pulmonary tuberculosis. Mil Med. 2005 Mar;170(3):211-3. | Only description of delay, not about the factors association with delay |
| 10 | Dong QL, Li YM, Wu DL, Guo HG, Wei CC. Delayed diagnosis or misdiagnosis of pulmonary tuberculosis: 458 cases clinical analysis. Di Yi Jun Yi Da Xue Xue Bao. 2004 Aug;24(8):943-5. | Only description of delay, not about the factors association with delay |
| 11 | Chern JP, Chen DR, Wen TH. Delayed treatment of diagnosed pulmonary tuberculosis in Taiwan. BMC Public Health. 2008 Jul 13;8:236. doi: 10.1186/1471-2458-8-236. | Only description of delay, not about the factors association with delay |
| 12 | Yilmaz A, Boğa S, Sulu E, Durucu M, Yilmaz D, Baran A, Poluman A. Delays in the diagnosis and treatment of hospitalized patients with smear-positive pulmonary tuberculosis. Respir Med. 2001 Oct;95(10):802-5. | Only description of delay, not about the factors association with delay |
| 13 | Chen TC, Lu PL, Lin WR, Lin CY, Lin SH, Lin CJ, Lo WC, Chen YH. Diagnosis and treatment of pulmonary tuberculosis in hospitalized patients are affected by physician specialty and experience. Am J Med Sci. 2010 Nov;340(5):367-72. doi: 10.1097/MAJ.0b013e3181e92b06. | Only description of delay, not about the factors association with delay |
| 14 | Behera BK, Jain RB, Gupta KB, Goel MK. Extent of delay in diagnosis in new smear positive patients of pulmonary tuberculosis attending tertiary care hospital. Int J Prev Med. 2013 Dec;4(12):1480-5. | Only description of delay, not about the factors association with delay |
| 15 | Verhagen LM, Kapinga R, van Rosmalen-Nooijens KA. Factors underlying diagnostic delay in tuberculosis patients in a rural area in Tanzania: a qualitative approach. Infection. 2010 Dec;38(6):433-46. doi: 10.1007/s15010-010-0051-y. Epub 2010 Sep 29. | Only description of delay, not about the factors association with delay |
| 16 | Yimer S, Holm-Hansen C, Yimaldu T, Bjune G. Health care seeking among pulmonary tuberculosis suspects and patients in rural Ethiopia: a community-based study. BMC Public Health. 2009 Dec 9;9:454. doi: 10.1186/1471-2458-9-454. | Only description of delay, not about the factors association with delay |
| 17 | Dhingra VK, Rajpal S, Taneja DK, Kalra D, Malhotra R. Health care seeking pattern of tuberculosis patients attending an urban TB clinic in Delhi. J Commun Dis. 2002 Sep;34(3):185-92. | Only description of delay, not about the factors association with delay |
| 18 | Auer C, Sarol J Jr, Tanner M, Weiss M. Health seeking and perceived causes of tuberculosis among patients in Manila, Philippines. Trop Med Int Health. 2000 Sep;5(9):648-56. | Only description of delay, not about the factors association with delay |
| 19 | Kapoor SK, Raman AV, Sachdeva KS, Satyanarayana S. How did the TB patients reach DOTS services in Delhi? A study of patient treatment seeking behavior. PLoS One. 2012;7(8):e42458. doi: 10.1371/journal.pone.0042458. Epub 2012 Aug 6. | Only description of delay, not about the factors association with delay |
| 20 | Wu YC, Hsu GJ, Chuang KY, Lin RS. Intervals before tuberculosis diagnosis and isolation at a regional hospital in Taiwan. J Formos Med Assoc. 2007 Dec;106(12):1007-12. doi: 10.1016/S0929-6646(08)60076-5. | Only description of delay, not about the factors association with delay |
| 21 | Mirsaeidi SM, Tabarsi P, Mohajer K, Falah-Tafti S, Jammati HR, Farnia P, Mansouri SD, Masjedi MR, Velayati AA. A long delay from the first symptom to definite diagnosis of pulmonary tuberculosis. Arch Iran Med. 2007 Apr;10(2):190-3. | Only description of delay, not about the factors association with delay |
| 22 | Yan F, Thomson R, Tang S, Squire SB, Wang W, Liu X, Gong Y, Zhao F, Tolhurst R. Multiple perspectives on diagnosis delay for tuberculosis from key stakeholders in poor rural China: case study in four provinces. Health Policy. 2007 Jul;82(2):186-99. Epub 2006 Oct 19. | Only description of delay, not about the factors association with delay |
| 23 | Wang WB, Jiang QW, Chen Y, Xu B. Pathways from first health care seeking to diagnosis: obstacles to tuberculosis care in rural China. Int J Tuberc Lung Dis. 2007 Apr;11(4):386-91. | Only description of delay, not about the factors association with delay |
| 24 | Qureshi SA, Morkve O, Mustafa T. Patient and health system delays: health-care seeking behaviour among pulmonary tuberculosis patients in Pakistan. J Pak Med Assoc. 2008 Jun;58(6):318-21. | Only description of delay, not about the factors association with delay |
| 25 | Edginton ME, Sekatane CS, Goldstein SJ. Patients' beliefs: do they affect tuberculosis control? A study in a rural district of South Africa. Int J Tuberc Lung Dis. 2002 Dec;6(12):1075-82. | Only description of delay, not about the factors association with delay |
| 26 | Okur E, Yilmaz A, Saygi A, Selvi A, Süngün F, Oztürk E, Dabak G. Patterns of delays in diagnosis amongst patients with smear-positive pulmonary tuberculosis at a teaching hospital in Turkey. Clin Microbiol Infect. 2006 Jan;12(1):90-2. | Only description of delay, not about the factors association with delay |
| 27 | Liu YC, Lin HH, Chen YS, Su IJ, Huang TS, Tsai HC, Wann SR, Lee SS. Reduced health provider delay and tuberculosis mortality due to an improved hospital programme. Int J Tuberc Lung Dis. 2010 Jan;14(1):72-8. | Only description of delay, not about the factors association with delay |
| 28 | Sabawoon Wrishmeen, Sato Hajime, Kobayashi YasukiPardis Ajmal. (2011) Regional differences in delay to tuberculosis treatment in Afghanistan: A cross-sectional study.Applied Geography 31: 1123-1131. | Only description of delay, not about the factors association with delay |
| 29 | Strand MA, Duan X, Johnson R, Li Y. Social determinants of delayed diagnosis of tuberculosis in a north China urban setting. Int Q Community Health Educ. 2010-2011;31(3):279-90. doi: 10.2190/IQ.31.3.f. | Only description of delay, not about the factors association with delay |
| 30 | Ohmori M, Ozasa K, Mori T, Wada M, Yoshiyama T, Aoki M, Uchimura K, Ishikawa N. Trends of delays in tuberculosis case finding in Japan and associated factors. Int J Tuberc Lung Dis. 2005 Sep;9(9):999-1005. | Only description of delay, not about the factors association with delay |
| 31 | Long NH, Diwan VK, Winkvist A. Difference in symptoms suggesting pulmonary tuberculosis among men and women. J Clin Epidemiol. 2002 Feb;55(2):115-20. | Only description of delay, not about the factors association with delay |
| 32 | Long NH, Johansson E, Lönnroth K, Eriksson B, Winkvist A, Diwan VK. Longer delays in tuberculosis diagnosis among women in Vietnam. Int J Tuberc Lung Dis. 1999 May;3(5):388-93. | Only description of delay, not about the factors association with delay |
| 33 | Mahendradhata Y, Syahrizal BM, Utarini A. Delayed treatment of tuberculosis patients in rural areas of Yogyakarta province, Indonesia. BMC Public Health. 2008 Nov 26;8:393. doi: 10.1186/1471-2458-8-393. | Only description of delay, not about the factors association with delay |
| 34 | Masjedi MR, Cheragvandi A, Hadian M, Velayati AA. Reasons for delay in the management of patients with pulmonary tuberculosis. East Mediterr Health J. 2002 Mar-May;8(2-3):324-9. | Only description of delay, not about the factors association with delay |
| 35 | Gosoniu GD, Ganapathy S, Kemp J, Auer C, Somma D, Karim F, Weiss MG. Gender and socio-cultural determinants of delay to diagnosis of TB in Bangladesh, India and Malawi. Int J Tuberc Lung Dis. 2008 Jul;12(7):848-55. | Definition of patient delay overlapped with definition of provider’s delay |
| 36 | Paul D, Busireddy A, Nagaraja SB, Satyanarayana S, Dewan PK, Nair SA, Sarkar S, Ahmed QT, Sarkar S, Shamrao SR, Harries AD, Oeltmann JE. Factors associated with delays in treatment initiation after tuberculosis diagnosis in two districts of India. PLoS One. 2012;7(7):e39040. doi: 10.1371/journal.pone.0039040. Epub 2012 Jul 9. | Definition of patient delay overlapped with definition of provider’s delay |
| 37 | Nasehi M, Hassanzadeh J, Rezaianzadeh A, Zeigami B, Tabatabaee H, Ghaderi E. Diagnosis delay in smear positive tuberculosis patients. J Res Med Sci. 2012 Nov;17(11):1001-4. | Definition of patient delay overlapped with definition of provider’s delay |
| 38 | Meyssonnier V, Li X, Shen X, Wang H, Li DY, Liu ZM, Liu G, Mei J, Gao Q. Factors associated with delayed tuberculosis diagnosis in China. Eur J Public Health. 2013 Apr;23(2):253-7. doi: 10.1093/eurpub/cks037. Epub 2012 Aug 8. | Definition of patient delay overlapped with definition of provider’s delay |
| 39 | Date J, Okita K. Gender and literacy: factors related to diagnostic delay and unsuccessful treatment of tuberculosis in the mountainous area of Yemen. Int J Tuberc Lung Dis. 2005 Jun;9(6):680-5. | Definition of patient delay overlapped with definition of provider’s delay |
| 40 | Cheng S, Chen W, Yang Y, Chu P, Liu X, Zhao M, Tan W, Xu L, Wu Q, Guan H, Liu J, Liu H, Chen RY, Jia Z. Effect of Diagnostic and Treatment Delay on the Risk of Tuberculosis Transmission in Shenzhen, China: An Observational Cohort Study, 1993-2010. PLoS One. 2013 Jun 27;8(6):e67516. Print 2013. | Definition of patient delay overlapped with definition of provider’s delay |
| 41 | Bam TS, Enarson DA, Hinderaker SG, Bam DS. Longer delay in accessing treatment among current smokers with new sputum smear-positive tuberculosis in Nepal. Int J Tuberc Lung Dis. 2012 Jun;16(6):822-7. doi: 10.5588/ijtld.11.0678. Epub 2012 Apr 9. | Definition of patient delay overlapped with definition of provider’s delay |
| 42 | Ahmad RA, Mahendradhata Y, Utarini A, de Vlas SJ. Diagnostic delay amongst tuberculosis patients in Jogjakarta Province, Indonesia is related to the quality of services in DOTS facilities. Trop Med Int Health. 2011 Apr;16(4):412-23. doi: 10.1111/j.1365-3156.2010.02713.x. Epub 2010 Dec 28. | Definition of patient delay overlapped with definition of provider’s delay |
