# Supplementary material for: Factors Associated with Patient and Provider Delays for Tuberculosis Diagnosis and Treatment in Asia: A Systematic Review and Meta-Analysis
Source: PLoS One. 2015 Mar 25;10(3):e0120088. doi: 10.1371/journal.pone.0120088 (PMC4373856; doi:10.1371/journal.pone.0120088)
Supplement: S3 Table — (DOC) [file pone.0120088.s003.doc]

**S3 Table. Results of subgroup analysis and test for heterogeneity of provider’s delay**

| **Variable** | **Endpoint** | **k** | **OR (95% CI) *** | **Heterogeneity between studies** | | | **Test for overall effect (P)** |
| --- | --- | --- | --- | --- | --- | --- | --- |
|  |  |  |  | **Q statistic** | **P-value** | **I^2^** |  |
| Consult with a public hospital | Diagnosis | 5 | 0.76 [0.59, 0.99] | 62.69 | <0.001 | 94% | 0.04 |
|  | Initial anti-TB treatment | 7 | 0.55 [0.47, 0.66] | 219.56 | <0.001 | 97% | <0.001 |
| Long travel time/distance to the first healthcare provider | Diagnosis | 5 | 1.68 [0.97, 2.91] | 28.01 | <0.001 | 86% | 0.06 |
|  | Initial anti-TB treatment | 2 | 1.64 [1.19, 2.25] | 0.43 | 0.51 | 0% | 0.002 |
| Age (older) | Diagnosis | 12 | 1.14 [0.70, 1.86] | 129.98 | <0.001 | 92% | 0.61 |
|  | Initial anti-TB treatment | 12 | 1.07 [0.98, 1.17] | 18.53 | 0.07 | 41% | 0.13 |
| Rural residence | Diagnosis | 4 | 1.16 [0.85, 1.58] | 7.96 | 0.05 | 62% | 0.34 |
|  | Initial anti-TB treatment | 4 | 1.87 [1.71, 2.04] | 45.83 | <0.001 | 93% | <0.001 |
| Income (low) | Diagnosis | 8 | 1.25 [0.84, 1.86] | 21.15 | 0.0007 | 72% | 0.27 |
|  | Initial anti-TB treatment | 2 | 0.71 [0.41, 1.21] | 0.00 | 0.98 | 0% | 0.20 |
| Unemployed | Diagnosis | 2 | 2.98 [0.75, 11.89] | 2.69 | 0.10 | 63% | 0.12 |
|  | Initial anti-TB treatment | 2 | 1.09 [0.79, 1.50] | 1.73 | 0.19 | 42% | 0.61 |
| Haemoptysis | Diagnosis | 5 | 0.73 [0.30, 1.77] | 44.76 | <0.001 | 91% | 0.48 |
|  | Initial anti-TB treatment | 3 | 0.86 [0.32, 2.31] | 7.45 | 0.02 | 73% | 0.77 |
| Positive smear | Diagnosis | 5 | 0.68 [0.07, 6.83] | 2124.59 | <0.001 | 100% | 0.74 |
|  | Initial anti-TB treatment | 3 | 0.53 [0.12, 2.35] | 72.79 | <0.001 | 97% | 0.40 |
| Chest radiographs | Diagnosis | 3 | 0.63 [0.19, 2.09] | 99.69 | <0.001 | 98% | 0.45 |
|  | Initial anti-TB treatment | 2 | 0.87 [0.19, 3.95] | 182.38 | <0.001 | 98% | 0.46 |
