# Supplementary material for: Factors Associated with Patient and Provider Delays for Tuberculosis Diagnosis and Treatment in Asia: A Systematic Review and Meta-Analysis
Source: PLoS One. 2015 Mar 25;10(3):e0120088. doi: 10.1371/journal.pone.0120088 (PMC4373856; doi:10.1371/journal.pone.0120088)
Supplement: S4 Table — (DOC) [file pone.0120088.s004.doc]

**S4 Table. List of all the scores for each item of the NOS of the included 45 studies**

| **Author, year** | **Selection** | **Comparability** | **Exposure** | **NOS score** |
| --- | --- | --- | --- | --- |
| Wang W (2007) | 3 | 0 | 1 | 4 |
| Leung EC (2007) | 3 | 0 | 1 | 4 |
| Huong NT (2007) | 3 | 1 | 1 | 5 |
| Enkhbat S (1996) | 3 | 0 | 1 | 4 |
| Yamasaki-Nakagawa M (2001) | 3 | 1 | 1 | 5 |
| Chiang CY (2005) | 3 | 1 | 1 | 5 |
| Rojpibulstit M (2006) | 2 | 1 | 2 | 5 |
| Li X (2012) | 3 | 1 | 1 | 5 |
| Lock WA (2011)26] | 3 | 1 | 1 | 5 |
| Zhou C (2012) | 3 | 1 | 1 | 5 |
| Chang CT (2007) | 3 | 1 | 1 | 5 |
| Lin X (2008) | 3 | 1 | 1 | 5 |
| Basnet R (2009) [30] | 3 | 1 | 1 | 5 |
| Guneylioglu D (2004) | 3 | 0 | 1 | 4 |
| Hoa NB (2011) | 2 | 1 | 2 | 5 |
| Xu B (2007) [33] | 2 | 0 | 2 | 4 |
| Ahmad RA (2013) [34] | 2 | 0 | 2 | 4 |
| Phoa LL (2005) [35] | 3 | 1 | 2 | 6 |
| Xu X (2013) [36] | 3 | 0 | 2 | 5 |
| Rundi C (2011) [37] | 3 | 1 | 2 | 6 |
| Zhao X (2014) [38] | 3 | 0 | 2 | 5 |
| Cheng G (2005) [39] | 2 | 1 | 2 | 5 |
| Tobe RG (2013) [40] | 3 | 1 | 2 | 6 |
| Choudhari M (2012) [41] | 3 | 1 | 2 | 6 |
| Shu W (2014) [42] ^a^ | 4 | 1 | 2 | 7 |
| Ngamvithayapong J (2001) [43] | 3 | 0 | 2 | 5 |
| Selvam JM (2003) [44] | 3 | 0 | 2 | 5 |
| Chen HG (2014) [45] | 3 | 1 | 2 | 6 |
| Kelkar-Khambete A (2008) [46] | 3 | 0 | 2 | 5 |
| Ayé R (2010) [47] | 2 | 0 | 2 | 4 |
| Tobgay KJ (2006) [48] | 3 | 1 | 2 | 6 |
| Rumman KA (2008) [49] | 3 | 1 | 2 | 6 |
| Wang Y (2008) [50] | 3 | 1 | 2 | 6 |
| Mor Z, (2013) [51] | 3 | 1 | 1 | 5 |
| Rifat M (2011) [52] | 3 | 0 | 2 | 5 |
| Bai LQ (2004) [53] | 3 | 1 | 2 | 6 |
| Tamhane A (2012) [54] | 3 | 0 | 2 | 5 |
| Sabawoon W (2012) [55] | 2 | 0 | 2 | 4 |
| Karim F (2007) [56] | 2 | 1 | 2 | 5 |
| Thakur R (2010) [57] | 3 | 0 | 2 | 5 |
| Lönnroth K (1999) [58] | 3 | 0 | 1 | 4 |
| Rajeswari R (2002) [59] | 3 | 1 | 1 | 5 |
| Lin HP (2009) [60] | 3 | 1 | 1 | 5 |
| Wang WB (2006) [61] | 2 | 0 | 2 | 4 |
| Lin CY (2010) [62] | 3 | 0 | 2 | 5 |

a, this study was a cohort study, so the questions were changed into representativeness of exposed cohort, selection of non-exposed cohort, exposure ascertainment, outcome not present at baseline, comparability of cohorts, outcome assessment, follow-up long enough, and follow-up adequate.
